# Frequency-dependent auditory space representation in the human planum temporale

**DOI:** 10.3389/fnhum.2014.00524

**Published:** 2014-07-22

**Authors:** Talia Shrem, Leon Y. Deouell

**Affiliations:** ^1^Human Cognitive Neuroscience Lab, Department of Psychology, Social Sciences Faculty, The Hebrew University of JerusalemJerusalem, Israel; ^2^Edmond and Lily Safra Center for Brain Sciences, The Hebrew University of JerusalemJerusalem, Israel

**Keywords:** sound location, adaptation, tonotopy, fMRI, sparse imaging

## Abstract

Functional magnetic resonance imaging (fMRI) findings suggest that a part of the planum temporale (PT) is involved in representing spatial properties of acoustic information. Here, we tested whether this representation of space is frequency-dependent or generalizes across spectral content, as required from high order sensory representations. Using sounds with two different spectral content and two spatial locations in individually tailored virtual acoustic environment, we compared three conditions in a sparse-fMRI experiment: Single Location, in which two sounds were both presented from one location; Fixed Mapping, in which there was one-to-one mapping between two sounds and two locations; and Mixed Mapping, in which the two sounds were equally likely to appear at either one of the two locations. We surmised that only neurons tuned to both location and frequency should be differentially adapted by the Mixed and Fixed mappings. Replicating our previous findings, we found adaptation to spatial location in the PT. Importantly, activation was higher for Mixed Mapping than for Fixed Mapping blocks, even though the two sounds and the two locations appeared equally in both conditions. These results show that spatially tuned neurons in the human PT are not invariant to the spectral content of sounds.

## Introduction

How space is represented in human auditory cortex is only rudimentarily known. Unlike the spatiotopic mapping of visual and tactile pathways, the primary organization of the auditory system is frequency based (tonotopic; Humphries et al., 2010; Striem-Amit et al., 2011). Nevertheless there is converging evidence from ferrets, cats and monkeys that within the auditory cortex, neurons located on the Planum Temporale (PT), on the posterior part of the superior temporal plane, are more narrowly tuned to the location of the sounds than other regions (Rauschecker and Tian, 2000; Recanzone, 2000b; Tian et al., 2001; Middlebrooks et al., 2002; Stecker et al., 2003; Woods et al., 2006; Miller and Recanzone, 2009). Congruently, in humans, fMRI and PET studies have found PT to be involved in spatial processing during active localization tasks (e.g., Zatorre et al., 2002; Zimmer and Macaluso, 2005; Zündorf et al., 2013), passive listening (e.g., Baumgart et al., 1999; Maeder et al., 2001; Warren and Griffiths, 2003; Brunetti et al., 2005; Krumbholz et al., 2005; Barrett and Hall, 2006), or even when the sounds are altogether ignored (Deouell et al., 2007). Thus, the PT is commonly regarded as a central stage of the dorsal, “where” pathway of the non-primary auditory cortex (Rivier and Clarke, 1997; Romanski et al., 1999; van der Zwaag et al., 2011) but whether it should be considered a “low level” or “high level” in the hierarchy of processing is unclear. A signature of high level representation of some feature is abstraction, that is, invariance to other features. Here we ask whether spatial tuning in the human PT is sensitive or invariant to the spectral content of sounds.

To that end, we applied the functional magnetic resonance adaptation (fMR-A) paradigm (Grill-Spector and Malach, 2001), which enables the characterization of the level of specificity vs. generality of neural populations within a voxel. Repetition of the same stimuli—or of stimuli that differ only on a dimension for which the neuron population within a voxel is invariant—causes a decrease in blood oxygen level dependent (BOLD) activation, which is interpreted as neural adaptation within these voxels. Thus, if presentation of stimuli that differ along a certain dimension leads to stronger activation than presentation of stimuli that vary less along this feature dimension, then neurons in that voxel are presumably sensitive to the dimension of variation. Along this rational, we examined BOLD activation in response to combinations of sounds of two different fundamental frequencies and two spatial locations. We surmised that if the same neurons in auditory regions are concurrently sensitive to both the spectrum and location of a stimulus (i.e., the location specificity is frequency-dependent), activation in these voxels would be higher for blocks in which all four combinations of frequency and location were presented than for blocks with fewer combinations. We show here that the spatial representation in the human PT is not invariant to the spectral content of the stimulus. That is, the same neurons are tuned to both frequency and spatial location of sounds.

## Materials and methods

### Subjects

Fifteen subjects (7 women), students at the Hebrew University of Jerusalem, age 24–33, (mean = 27.93, std = 2.76), 4 left-handed, participated in the experiment. Two additional subjects were excluded, one for not being able to localize the sounds in the scanner (the subject was not scanned as she could not localize the sounds in the preliminary location discrimination task), and one due to substantial head movements during the experiment. They all had normal, symmetrical hearing, according to their report. All subjects were screened for MRI compatibility and gave informed consent prior to participation. Subjects were paid for their time. The procedures were approved by the ethics committee at the Loewenstein Rehabilitation Center, Raanana, Israel, and at the Hebrew University of Jerusalem.

### Stimuli

**Figure 1:**
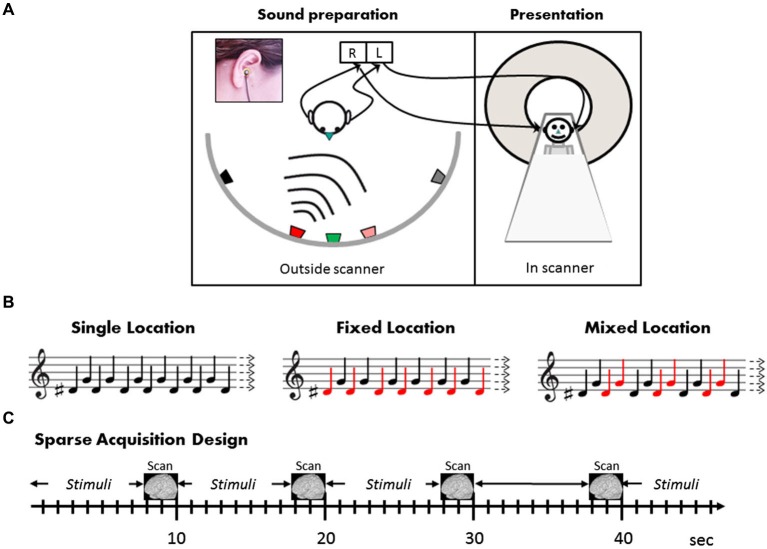
**Stimuli and design**. **(A)** Outside the scanner, the subject (left panel) was seated in the center of a semicircular array of five loudspeakers positioned 90 cm from the center of the head at ±60°, ±15°, and 0° relative to the midsagittal plane. Two miniature microphones embedded in standard ear plugs were placed in the external auditory canals, pointing outwards, with their front end aligned with the external auditory meati (inset). In the scanner, the individually tailored sounds thus recorded were then presented to subjects (right panel) by earphones. **(B)** Illustration of the stimulation conditions (see text for details). Note that in all conditions, half the sounds in each block were “high” (*F*_0_ = 784 Hz, G note) and half “low” (*F*_0_ = 622 Hz, D#). The difference between the sound blocks is in the mapping between high and low (illustrated by the musical notes) and the two sound locations (depicted by red and black notes). The subjects watched a movie and were instructed to ignore the sounds. **(C)** The three conditions as well as silence blocks were presented in pseudo-random order in a sparse acquisition design. A single EPI volume was acquired in 2.29 s, and 20 sound stimuli were presented within 7.71 intervals between scans, i.e., with no interruption of scanning noise.

The auditory stimuli were prepared individually for each subject in a separate recording session prior to the fMRI experiment. The subject was seated in a sound-attenuated and echo-reduced chamber, in the center of a semicircular array of five loudspeakers (Peerless model 821615 midrange 122M) positioned at approximately ear height, in the frontal plain, 90 cm from the center of the head at ±60°, ±15°, and 0° relative to the midsagittal plane (negative numbers refer to the left hemifield) (Figure 1A). Two miniature electret microphones (Sennheiser KE4-211-2) embedded in standard E.A.R. foam ear plugs were placed in the external auditory canals, pointing outwards, with their front end aligned with the external auditory meati (see Deouell et al., 2007). As simple tones are hard to localize, the two auditory stimuli comprised of 200 ms complex tones with a fundamental of 622 Hz or 784 Hz, and including equal amplitude overtones of 1.5, 4.5, 6 times the fundamental frequency, amplitude modulated sinusoidally at 50 Hz. The stimuli were created digitally in Cool Edit 2000 software [Synrillium] at 44.1 KB/16 bits. While the subject’s head pointed straight ahead, each of the two sounds was played from each loudspeaker in turn, with 800 ms inter-stimulus interval, and recorded by the intra-aural microphones via a stereophonic preamplifier (DMP3 preamplifier, M-Audio, USA; Audigy 2 ZS sound card; Creative, USA; 16 bits; 44.1 Kb/sec). Recorded this way, the stereophonic sounds genuinely reflected the sound pressure at the external auditory meati of the subjects, allowing all individual binaural and pinnae related spatial cues to be saved (Møller et al., 1996; Hammershoi and Moller, 2002). The recorded sounds were high-pass filtered at 125 Hz to remove electric hum, then trimmed to 250 ms segments, including 50 ms after the stimulus offset, encompassing room reverberations extending after the offset of the played sound. These individual binaural recordings were used as subject-specific stimuli in the fMRI experiment.

### Design and procedure

While in the scanner, the subject-specific auditory stimuli were played to the subject via MR compatible electrodynamic headphones (MR-Confon).

Once the subject was positioned in the scanner, and prior to the scanning, the effectiveness of the individualized virtual spatial locations was validated using a sound location discrimination task. Subjects then underwent a T1 weighted coplanar anatomical scan, followed by eight T2*-weighted echo-planar imaging (EPI) functional scans, and a high-resolution 3D MPRAGE T1 weighted image of 1 × 1 × 1 mm.

In the location discrimination task, subjects were presented with 36 pairs of sounds, taken from their individually recorded +15°, +60°, −15°, −60° sounds. In each pair, the two sounds could be either from the same location, or from the other location within the same hemispace, so that the subjects could not rely on simple lateralization of sounds to right or left of the midline (for example, if the first sound was at 15° the second sound was either at the same location or at 60°). Following each pair presentation subjects were required to state whether the second sound was presented from the left, from the right, or from the same location as the first. Each of the three responses had the same probability, and the pairs were presented in random order. Responses were collected by using three buttons of a response box. This was done both for the higher pitch and for the lower pitch sounds. For each pitch, two of the recorded sounds, both within the right hemispace (+15°, +60°) were then used as stimuli in the main experiment.

Following the location discrimination task, subjects completed eight fMRI runs of 32 × 10 s blocks each, in a sparse block design (Hall et al., 1999). In this sparse design, auditory stimuli were played within 7.71 s silent periods, that is, without interfering scanner noise. Stimulus presentation was followed by a clustered volume acquisition (see below). The next block of stimuli started immediately after the end of the previous acquisition. Stimulation was synchronized with the scanner via TTL pulses sent by the scanner at the onset of each volume acquisition. The subjects’ only task was to watch a subtitled silent movie and ignore the background sounds (experimental sounds and scanner noise). Different combinations of two sounds (“low”—F0 = 622 Hz and “high”—F0 = 784 Hz) from the two right hemispace locations (15°, 60°) were presented in separate blocks, creating three experimental conditions (Figure 1B): Single Location, Fixed Mapping, and Mixed Mapping. Only one hemispace was tested to allow enough repetitions considering the sparse design, within a reasonable scanning session. Notably, in all three experimental conditions the same balanced composition of low- and high-frequency sounds were present, and the blocks differed in the number of locations (one or two), and in the mapping of pitch to location. In the Single Location blocks, the low and high sounds were presented in alternation both from the same location (i.e., only one location was present in this block). Half of the Single Location blocks were at the more medial and half at the more lateral location. In the Fixed Mapping blocks, the low and high sounds were presented from the two locations, and each location was consistently mapped to one of the two sounds, creating two frequency-location combinations which alternated, whereas in the Mixed Mapping blocks, each of the two sounds was equally likely to be presented from either location, creating four frequency-location combinations (the two frequencies again alternated, while the two locations alternated in an AABBAABB… pattern; see Figure 1). In a fourth control condition (Silence blocks) no sounds were presented. Each block (except Silence) was composed of 20 equally-spaced stimuli (400 ms onset-to-onset). Each condition was presented in eight blocks per run (32 blocks/run altogether). The order of the blocks was quasi-random ensuring no immediate repetition of the same type of block, and no clustering of one type of block within the run. To ensure the functionality of the sound delivery system, at the beginning of each run subjects were presented with the four sounds and were asked whether they heard them properly.

This design enabled us to assess the hypothesis that sensitivity to stimulus location in the auditory cortex interacts with sensitivity to the spectral content within a region sensitive to auditory spatial location. First, we expected blocks with a single location to induce stronger adaptation (and hence lower BOLD signal) in the PT than blocks in which two locations are presented (Fixed and Mixed Mapping blocks). This would replicate our previous results (Deouell et al., 2007) and create the functional region of interest (ROI) for the critical contrast involving the Fixed and Mixed Mapping blocks: if location sensitive neurons are invariant to sound frequency, the same amount of adaptation (hence similar BOLD signal) should be exhibited in these two kinds of blocks. Conversely, if the same neurons are sensitive to both spectrum and location, lower BOLD activation (due to more adaptation) is expected in response to Fixed Mapping blocks than to Mixed Mapping blocks. In addition to the data driven ROI, defined as location sensitive voxels in the STG (for the sake of defining an ROI, Mixed Mapping and Fixed Mapping were treated as one, since both conditions consisted of sounds from the two locations), an *a-priori* ROI was applied from our previous study using a similar contrast (Deouell et al., 2007).

### MRI data acquisition

Scanning was performed on a Siemens 3 Tesla Trio scanner, at the Asher Center for Human Brain Imaging at the Weizmann Institute, Rehovot, Israel. Thirty five 4 mm slices aligned with the Sylvian fissure and covering the whole brain were acquired in an interleaved order of acquisition, with in plane resolution of 3 × 3 mm. EPI sequence parameters: flip angle = 90°, TE = 30 ms, TR = 10 s. Using a sparse design with clustered volume acquisition (Hall et al., 1999), scanning was clustered within 2.29 s time of acquisition (TA) at the last part of each 10 s TR, leaving the rest of the time silent for uninterrupted presentation of sounds (Figure 1C). The functional scans were preceded by a T1 coplanar anatomical scan and followed by a high-resolution 3D MPRAGE T1 weighted image of 1 × 1 × 1 mm.

### fMRI data analysis

fMRI analysis was performed using SPM2.^1^ The first EPI image of each scan was removed, and scans were motion corrected by realigning to the second EPI image of each scan. Images were smoothed with an 8 × 8 × 8 Gaussian kernel and normalized to the MNI space with a resolution of 2 × 2 × 2 mm. Stimulation-related activation was mapped at the single subject level using the general linear model (GLM) approach. Each block was modeled as an event of 8 s duration, convolved with a canonical HRF. Low-frequency noise and linear trends were removed from each run, using a high-pass filter with a cutoff of 0.008 Hz.

To create a functional ROI consisting of voxels showing location sensitivity, we ran a second order group random effect analysis contrasting the pooled Mixed and Fixed parameter estimates (betas) against Single Location betas, confined to the STG as defined by Automated Anatomical Labeling system (AAL; Tzourio-Mazoyer et al., 2002), encompassing the “superior temporal gyrus”, “Heschl gyrus” and the “temporal pole” areas, bilaterally. This was statistically thresholded at *p* < 0.05, corrected for multiple comparisons using the AFNI Alphasim algorithm as implemented in the REST toolbox for Matlab (Song et al., 2011). This algorithm implemented a Monte Carlo simulation with 1000 iterations to determine the maximal extent of clusters of voxels (rmm setting = 5), each passing an uncorrected threshold of *p* < 0.01, that would be found by chance, under the null hypothesis of no difference (cf. Forman et al., 1995; Xiong et al., 1995). To test our major question regarding combined sensitivity of frequency and location, we performed a second order random effects group analysis contrasting the Mixed Mapping condition vs. the Fixed Mapping condition. We then tested whether the mean beta values of this contrast, within the above functional ROI, were significantly larger than 0, using Student’s *t*-test.

In addition, to rule out any effect of dependency between our functional definition of ROI and the contrast of interest (which were recorded in the same experiment), we adapted an ROI of location sensitive voxels from Deouell et al. (2007), and repeated the analysis using this predefined ROI. This ROI was defined in Deouell et al. (2007) as voxels showing higher activation to blocks including location deviant sounds than to blocks including only one location, confined to the AAL-defined STG region (see above) (*p* < 0.001, uncorrected, with a minimal cluster size of 10 voxels). This is the same ROI used in experiments 2 and 3 of Deouell et al. (2007).

## Results

### Sound localization

In the location discrimination task all 15 subjects were able to discriminate the virtual locations of sounds properly with an above 85% success rate. Mean accuracies were 97.6% (std = 4.41) and 94.21% (std = 4.51) for high pitch (784 Hz) and low pitch (622 Hz) sounds respectively. No significant difference was found between accuracy for high and low pitch sounds.

### BOLD analysis

**Figure 2:**
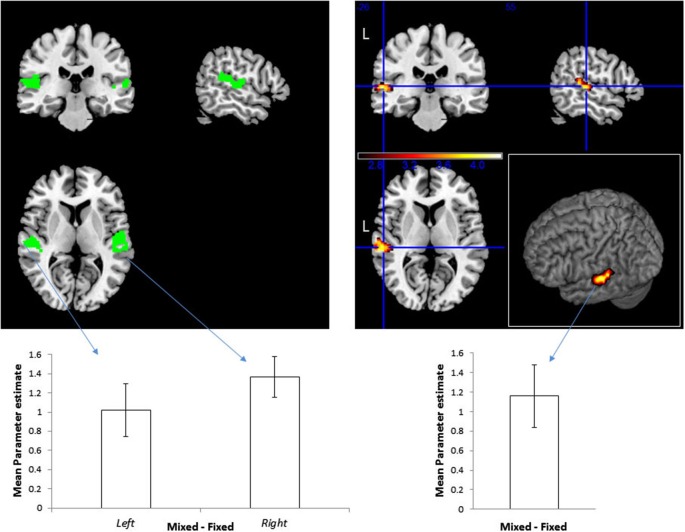
**Effect of the frequency and sound location combinations on BOLD signal**. Right: Top—Functional ROI of location-sensitive voxels in the STG, specified by contrasting Mixed Mapping + Fixed Mapping vs. Single Location blocks, *p* < 0.05, corrected. The color scale represents *t*-values. Bottom—mean (and standard error of the mean) of beta values difference within this ROI for the contrast Mixed > Fixed. Left: Top—Pre-defined ROI of location sensitive voxels within the STG, based on an independent set of subjects from Deouell et al. (2007). Bottom—mean beta values difference for the contrast Mixed > Fixed within the ROIs, and standard errors of the means within this ROI.

To define location-sensitive voxels, we contrasted the blocks with spatial variation (including the Fixed and Mixed Mapping conditions) against the blocks without spatial variation (the Single Location blocks). Replicating our previous study (Deouell et al., 2007), this resulted in a cluster of significant activity within the PT of the left hemisphere (Figure 2; peak [*x*,*y*,*z*] MNI coordinates = [−60, −26, 4]; note that the sounds were spatially localized to the right). This cluster was used as an unbiased “functional ROI” to test our main question—whether location—sensitive neurons in the auditory cortex are selective to combinations of sound pitch and location—by contrasting the Mixed against the Fixed Mapping condition. We reasoned that for spatially selective neurons which are invariant to the spectral content of the sounds, the Fixed and Mixed conditions should be identical (the spectral variation *per se* was similar in all blocks, as was the spatial variation). In contrast, for spatially selective neurons that are also selective for the spectral content, neurons should be less adapted in the Mixed condition than in the Fixed condition, showing higher overall activity. The results confirmed the latter option: the activity in the Mixed Mapping blocks was significantly stronger than in the Fixed Mapping (one-tailed *t*_(14)_ = 3.61, *p* = 0.0014).

Although the procedure for selection of our functional ROI could not bias the result of the main contrast of Fixed vs. Mixed Mapping, we repeated the analysis using an *a-priori* ROI derived from the results of Deouell et al. (2007), experiment 1, contrasting sequences with spatial deviation against stimuli presented from a fixed location. This is the identical ROI that was used in Deouell et al.’s Experiment 2 and 3. Here, we first used this ROI to test whether the sensitivity to spatial location will be replicated. Indeed, the contrast between the two conditions with spatial variation (Fixed and Mixed Mapping pooled) and the condition without spatial variation (Single Location) was significant on the left STG (one-tailed one-sample *t*_(14)_ = 2.6687, *p* = 0.0092) and marginally significant on the right (*t*_(14)_ = 1.7282, *p* = 0.053). Next, we contrasted the Mixed against the Fixed Mapping conditions within this *a-priori* location-sensitive ROI. The activity in the Mixed Mapping blocks was stronger than in the Fixed Mapping blocks, both on the left (*t*_(14)_ = 3.6869, *p* = 0.0012, one-tailed), and on the right (*t*_(14)_ = 6.4949, *p* = 8.34*10^−6^).

## Discussion

Arguably, the goal of the auditory system is to determine “what made that sound”, “what does it mean” and “where did this sound come from?” However, answering these questions comes on unequal footage. The first two depend heavily on the frequency content of the sounds, which is directly represented, in a tonotopic organization, starting from the sensory epithelium (the basilar membrane). In contrast, the spatial source has to be computed based mainly on the difference between the frequency-specific inputs to the two ears. The present study addressed the way the spectral and spatial dimensions of auditory stimuli interact in auditory cortex.

Previous studies have pointed to the PT as harboring the sharpest spatial representation amongst the non-primary auditory areas (e.g., Baumgart et al., 1999; Rauschecker and Tian, 2000; Recanzone, 2000b; Maeder et al., 2001; Tian et al., 2001; Middlebrooks et al., 2002; Pavani et al., 2002; Stecker et al., 2003; Warren and Griffiths, 2003; Smith et al., 2004; Brunetti et al., 2005; Krumbholz et al., 2005; Barrett and Hall, 2006; Woods et al., 2006; van der Zwaag et al., 2011). Using an fMRI adaptation approach (Grill-Spector et al., 2006; Krekelberg et al., 2006) the current study directly challenged the possibility that this spatial representation is “high level”, in the sense that it represents the spatial location of the sounds independent of their spectral content (i.e., demonstrates spectral invariance or generalization). Mixed Mapping blocks were contrasted against Fixed Mapping blocks; while the same locations and frequencies were presented equally in both conditions, the Mixed Mapping blocks consisted of all four combinations of location and frequency (two locations × two frequencies), whereas the Fixed Mapping blocks consisted of only two combinations in a given block. Thus, the only difference between the two conditions is the number of specific associations of locations and frequency, and therefore spatially selective neurons that are indifferent to the spectral content should be equally adapted in the two mapping conditions. Differences in activation between these mapping conditions can arguably result only from the presence of populations of neurons that are simultaneously tuned to location and pitch. Such neuronal populations would be less adapted in the Mixed Mapping condition than in the Fixed Mapping condition, as each population is triggered less frequently in the former condition than in the latter. Whether using an *a-priori* functional ROI or a within-experiment functional ROI, we find that spatial location, at a within-hemispace resolution, interacts with spectral content in eliciting BOLD activation in the PT, a major starting point of the auditory “dorsal” pathway.

Similar to the division found in visual processing (Mishkin et al., 1983) spatial location and other characteristics of sound seem to be conveyed in anatomically separate dorsal and ventral pathways, respectively (Rauschecker, 1998; Romanski et al., 1999; Rauschecker and Tian, 2000; Alain et al., 2001; Tian et al., 2001; Clarke et al., 2002; Zatorre et al., 2002; Viceic et al., 2006). Notably, the “where” stream presumably starts at the PT, in areas that are known in monkeys as CL and CM of the caudal belt (Rauschecker and Tian, 2000; Recanzone, 2000b, 2001; Kuśmierek and Rauschecker, 2014) and in humans as LA, PA and STA (Rivier and Clarke, 1997; van der Zwaag et al., 2011). However, the distinction between processing of spatial and spectral features of sounds might not be complete.

In addition to spatial tuning, neurons in monkey CM and CL show some spectral tuning (Recanzone, 2000a; Tian et al., 2001), and even a degree of tonotopic organization, which was in fact the basis for their delineation from other regions (Kosaki et al., 1997; Rauschecker and Tian, 2004; Petkov et al., 2006; Kuśmierek and Rauschecker, 2014). Moreover, the response of at least some neurons in area CL of the monkey may co-vary with both spectro-temporal content and spatial location (Tian et al., 2001, see also Ma et al., 2013). Overlap between spatial and non-spatial information was also found in more downstream parts of both pathways in the monkey (e.g., Cohen et al., 2004). Although intrinsically crude, human EEG source analysis also suggested that spatial information may be present not only in the dorsal but also in the ventral auditory pathway (Bourquin et al., 2013), perhaps later in time than in the dorsal stream (Lewald and Getzmann, 2011). Supporting the overlap, task irrelevant pitch differences facilitate near-threshold location discrimination, and vice versa (Tardif et al., 2008), and adaptation to a lateralized stimulus involves frequency specific channels (Phillips and Hall, 2005; Vigneault-MacLean et al., 2007; Stange et al., 2013). However, the anatomical extent of this frequency-space interaction, especially in humans, is not known (see Stange et al., 2013 for evidence for frequency specific spatial adaptation as early as the Medial Superior Olivary [MSO] nucleus of the Gerbil, and Brown et al., 2012 for an even earlier, pre-binaural site in humans). Moreover, evidence for independent representations of space and frequency have also been found in humans.

Schröger (1995) and more recently Du et al. (2011) investigated the possible interaction between space and pitch representation using the mismatch negativity (MMN) event-related potential,^2^ and found that the responses to changes in the spectral content and in the location of sounds, were additive. This suggested independent, rather than interactive, representation of the two dimensions in the auditory cortex, which is the major source of the MMN. Inconclusive behavioral results were obtained using the “ventriloquism after-effect”. The ventriloquism effect is formed when subjects are exposed to pairs of a visual stimulus and a tone with some spatial discrepancy between them. The perceived location of the sounds is typically shifted towards the location of the visual stimulus (for review see Chen and Vroomen, 2013). Moreover, subsequent tones which are then presented without the visual stimulus are also perceived as shifted (“ventriloquism after-effect”), suggesting a recalibration process (Recanzone, 1998). While some find that this after-effect pertains only to the frequency of the tone of the trained audio-visual pair, suggesting frequency-dependent spatial representation, others find that it affects tones of other, distinct frequencies, suggesting frequency invariant spatial representation (Recanzone, 1998; Lewald, 2002; Frissen et al., 2003, 2005; Woods and Recanzone, 2004). A recent computational model suggested that the test sound intensity may determine the amount of spectral generalization in this paradigm (Magosso et al., 2013).

Interaction between location and spectro-temporal pattern (represented by different animal vocalizations) within the location-selective left PT has previously been reported using human fMRI by Altmann et al. (2008), who used an event-related variation of the fMRI adaptation approach. Pairs of same or different animal vocalizations were presented from the same or different location, and the degree of (release from) adaptation was assessed. Relevant to the present study was the fact that the effect of spatial change and pattern (vocalization) change interacted (under-additively), suggesting mutual representation of space and spectro-temporal information. However, this study was designed to test the effect of feature specific attention, and therefore subjects performed either a location-discrimination or pattern-discrimination task on each pair. As noted above, location information and spectral information interact behaviorally (Tardif et al., 2008; and also in Altmann et al., 2008), and therefore the interaction observed at the BOLD level could be due to top-down or performance-related effects rather than bottom-up effects reflecting neuronal tuning. Moreover, the complexity and semantic content of the stimuli makes it difficult to pinpoint the relevant dimension interacting with location. The present data, obtained when subjects were occupied with a visual task, and with meaningless sounds differing in spectrum (and therefore in pitch) provides direct evidence that at least at the early stages of the auditory dorsal stream in the human PT, spatial information is not invariant to spectral content.

**Figure 3:**
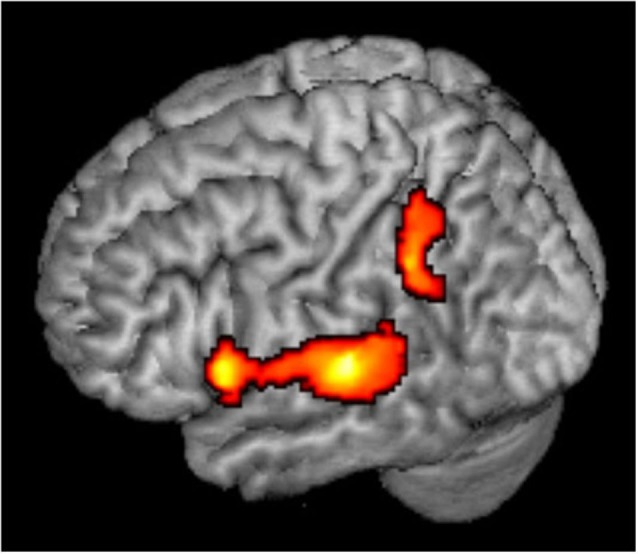
**Whole brain analysis**. Mixed Mapping > Single Location contrast, showing significant voxels in color; *p* < 0.05, corrected.

Rather than being a high level spatial node, the PT may serve as an intermediate station, perhaps as a computational hub (Griffiths and Warren, 2002), which segregates spatial from non-spatial features of sound and transmits the different types of information to further processing in the ventral and dorsal streams. Evidently, the present results suggest that within the PT, the information has not been completely segregated yet. It is also possible that while individual spatially selective neurons maintain their frequency selectivity, a spatial code emerges at the population level, and this code is invariant to other features (Miller and Recanzone, 2009; Lüling et al., 2011). This frequency-invariant population code may be read out in higher order regions of the auditory dorsal stream. The present data can only hint to these higher order regions (Figure 3). When the least adapted condition (Mixed Mapping) was contrasted with the most adapted (Single Location), not only bilateral PT showed spatial sensitivity, but also left inferior parietal cortex and left inferior frontal cortex (peak [*x*,*y*,*z*] MNI coordinates = [−46, −44, 36], [−57,14,6], respectively, cf. Deouell et al., 2007; At et al., 2011). However, there was no significant difference in the activity between Mixed and Fixed Mapping in these extra-temporal regions, suggesting spectral invariance. This conclusion should be taken with caution though, as it is based on the latter null result.

Could the difference between the mixed and the fixed conditions result from attention being diverted towards the auditory stream in the Mixed Mapping condition more than in the Fixed Mapping condition? In a previous study, we addressed the PT activation related to spatial change and compared a condition in which subjects watched a silent movie (as done here) with a condition in which they performed a demanding visual go-nogo task (Deouell et al., 2007). We found no evidence for an effect of the level of attention required by the visual task on the level of activation in PT. In the present study subjects again watched a silent movie and ignored the sounds. In all conditions, the frequency of the sounds alternated, and thus the streams sounded overall quite similar. Moreover, in the critical Mixed and Fixed conditions, the same two frequencies and locations were presented, making the streams sound highly similar. Furthermore, each condition was presented many times, was never task relevant, and there were no salient events in any sequence which would attract the subjects’ attention. Therefore, we find it highly unlikely that the subjects directed more attention to the sounds in the Mixed condition than in the Fixed condition. Similarly, it is unlikely that the response in the Mixed condition was driven by a type of mismatch response (akin to the MMN) because of the switching between pairs of locations in the Mixed condition. First, we are not aware of evidence for a mismatch negativity following minimal (*n* = 2) sequences of stimuli (especially with repeating locations) in a regular fast design. Second, we previously showed (Deouell et al., 2007, Experiment 2) that relative to a repetitive single location condition (the Single condition) a sequence with much more potential to elicit a mismatch response (an AAAABAAA…sequence with rare deviants) does not elicit a larger effect than an alternating ABAB sequence (called Equal condition in Deouell et al., 2007, resembling the Fixed condition in the current study) in this region.

The current results support our previous fMRI findings (Deouell et al., 2007) showing that neurons in the human PT are sensitive to within-hemispace sound location, even when the task does not require attention to the location of sounds nor to sounds in general. Using the MMN event-related brain potential in humans, we previously demonstrated that neurons in this region may resolve as little as 10° of spatial deviation (Deouell et al., 2006) and that it may represent space in a head-independent manner, suggesting integration of acoustic information with information about head-on-trunk position (Schechtman et al., 2012; but see different results in Altmann et al., 2009, 2012). Interestingly, a recent study in the anesthetized cat *primary auditory cortex* suggests that in the context of sounds presented from multiple locations, spatial tuning of neurons becomes sharper than previously found with single location streams, yielding a resolution at or below 10° (Middlebrooks and Bremen, 2013). Under the assumption that the same mechanism can be seen in the PT, this suggests that the comparison between single location conditions and multiple (Fixed or Mixed) conditions involves not only release from adaptation, but also a change in the width of the tuning curve of neurons.

In summary, the data presented here bolster the involvement of the PT in pre-attentive spatial representation in humans and shows that spatially selective neurons in the PT are not invariant to the frequency content of the sounds. Considering that sound frequency is the main organizing dimension in audition, this maintenance of frequency channels may be comparable to the maintenance of retinotopy (i.e., location sensitivity, the primary organizing dimension of vision) recently found in downstream regions of the visual cortex (see Wandell and Winawer, 2011 for review).

## Conflict of interest statement

The authors declare that the research was conducted in the absence of any commercial or financial relationships that could be construed as a potential conflict of interest.

## Footnotes

^1^http://www.fil.ion.ucl.ac.uk/spm/software/spm2

^2^Mismatch negativity is a pre-attentive index of sound-features representation in the auditory cortex, peaking 100–200 ms after sound onset, in response to unattended deviants in a sequence of sounds.
